# Resistance exercise training lowers HbA1c more than aerobic training in adults with type 2 diabetes

**DOI:** 10.1186/1758-5996-1-27

**Published:** 2009-12-10

**Authors:** Salameh Bweir, Muhammed Al-Jarrah, Abdul-Majeed Almalty, Mikhled Maayah, Irina V Smirnova, Lesya Novikova, Lisa Stehno-Bittel

**Affiliations:** 1 Department of Physiotherapy, Allied Medical Sciences, Hashemite University, Zarqa, Jordan; 2 Department of Physiotherapy, Applied Medical Sciences, Jordan University of Science and Technology, Irbid, Jordan; 3 Department of Physical Therapy and Rehabilitation Science, University of Kansas Medical Center, Kansas City, KS, USA

## Abstract

**Background:**

The aim of this study was to compare the effects of 10 weeks of resistance or treadmill exercises on glycemic indices levels prior to and immediately following exercise in adults with type 2 diabetes.

**Research Design and Method:**

Twenty inactive subjects (mean age 53.5 years) with type 2 diabetes enrolled in the study. Baseline HbA1c, blood glucose levels, heart rate, and blood pressure were measured for each subject prior to the initiation of the exercise program. Subsequently, subjects were matched to age, waist circumference and sex and assigned to either isocaloric resistance or treadmill exercise groups, which met 3 times per week for 10 weeks.

**Results:**

Both groups showed a reduction in pre and post-exercise blood glucose and HbA1c values. There was no change in resting blood pressure or heart rate in either group during the course of the 10 week intervention. The group receiving resistance exercises showed significant differences in the daily pre-exercise plasma glucose readings between the beginning and end of the exercise protocol (p < 0.001). There were significant improvements in the mean HbA1c reading pre and post training in both groups (p < 0.001). However, the greater reduction was noted in the resistance exercise group, and at 10 weeks their HbA1c levels were significantly lower than the group that received treadmill exercises (p < 0.006).

**Conclusion:**

Ten weeks of resistance exercises were associated with a significantly better glycemic control in adults with type 2 diabetes compared to treadmill exercise.

## Introduction

Strict control of hyperglycemia is an essential part of the management of diabetes in order to prevent chronic progression of the complications. Several large-scale trials have shown that microvascular complications can be positively affected by tight glycemic control [1-4]. Alternatively, the effect of glycemic control on macrovascular complications is more controversial [5,6], including findings of the ACCORD study indicating that glycemic control is associated with a higher rate of mortality [7,8]. Most large-scale studies have utilized pharmacological interventions to achieve glucose control [6,9,10]. Given the controversy surrounding pharmacological interventions to maintain tight glycemic control in people with type 2 diabetes [9], it is important to examine alternative approaches with potentially less risk of mortality.

Several studies, including meta-analysis work, have confirmed the beneficial effect of exercise training on glycemic control [11,12] and blood pressure [13]. The current guidelines from the American Diabetes Association, the European Association for the Study of Diabetes, and the American Heart Association recommend regular aerobic and resistance exercise for people with type 2 diabetes without major complications [14,15]. However, the guidelines are written to be extremely general and they do not provide information on the intensity or most beneficial type of exercise for specific subpopulations of people with diabetes in order to maximize the benefit, while maintaining minimal risk [12,15,16].

In choosing any exercise program, one of the first questions to be examined should be the impact of aerobic versus resistance exercises. Specifically, aerobic exercise training has been shown to improve glycemic control, insulin sensitivity and VO_2_max [12]. In addition, the more intense the aerobic exercise the better the glycemic control and insulin sensitivity [17-19].

Resistance exercise has only recently received attention for its effects on glycemic control in people with diabetes. In fact, a 2009 review of publications concerning resistance exercise and changes in metabolic outcomes found only 9 peer-reviewed papers [20]. The studies showed mixed results concerning the ability of resistance exercises to improve the metabolic abnormalities associated with diabetes (see review by [21]). Baldi and Snowling found that resistance training failed to alter hemoglobin A1c (HbA1c) levels significantly [22]. However, other studies concluded that glycemic control could be improved with resistance training [23-26]. In fact, Ishii et al. [23] showed that the glucose disposal rate was nearly double following resistance training of 4-6 weeks, but HbA1c levels did not change. More recent studies have reported significant declines in HbA1c with resistance training [24-26], possibly by improving the storage and utilization of glucose in muscle [12,27].

Only a few studies have directly compared the effects of resistance to aerobic exercise for people with diabetes in the control of blood glucose levels. Snowling et al. found that both resistance and aerobic exercise appeared to control blood pressure in people with diabetes to a similar extent [28]. Cauza et al. found that resistance exercises were as good or even more beneficial than aerobic training in maintaining blood glucose levels [29]. In contrast Sigal et al. reported that combined aerobic and resistance training was superior to either alone [30]. The purpose of this study was to compare aerobic to resistance exercise protocols in a matched subject population while controlling for the intensity of the exercise protocols.

## Research Design and Methods

### Subject characteristics

Twenty-three previously inactive patients between 45 and 65 years of age with type 2 diabetes were recruited from outpatient diabetes clinics of the AL Basheer General Hospital in Amman, Jordan. Inclusion and exclusion criteria are shown in Table 1. Subjects with diabetes-related complications that prohibited participation in exercise were excluded from the study. The characteristics of the subjects in the two groups are shown in Table 2. The study was approved by the Institutional Human Subjects Committee.

**Table 1:** Inclusion and exclusion criteria for participation in the study

Eligibility Criteria	Exclusion Criteria
Cleared by a general practitioner to attend the program	Current insulin therapy
Type 2 diabetes for more than 6 months	Participation in exercise 2 or more times weekly for 20 minutes or longer per session or in any resistance training during the previous 6 months
A baseline HbA1c value of 7 to 10.5	Changes during the previous 2 months in: oral hypoglycemic medication antihypertensive medication lipid-lowering medication body weight (≥ 5%)
Able to regularly attend the training programs for 3 sessions per week for over 10 weeks	Blood pressure greater than 160/95 mm Hg
	Restrictions in physical activity because of disease; or presence of other medical conditions that made participation inadvisable

**Table 2:** Characteristics of participants prior to exercise intervention

Characteristics	Resistance exercise (n = 10)	Treadmill exercise (n = 10)
Age (years)	53.4 ± 7.2	53.4 ± 10.2
Male	8	9
Female	2	1
Blood pressure (mm Hg)	138 ± 2/85 ± 1	135 ± 2/84 ± 1
Heart rate (beats/minutes)	86 ± 1	85 ± 1
Waist Circumference (cm)	93 ± 7	96 ± 6
HbA1c (%)	8.8 ± 1.1	8.7 ± 0.7

### Study description

A 10 week exercise intervention study was conducted using a single-center, controlled trial with parallel-group design and matched subjects. Initially subjects were monitored for a 12 week pre-intervention period in order to monitor glycemic control and changes in medications prior to the initiation of the exercise intervention. During this control period, subjects were asked to continue with their normal life and regular medications and diet. The HbA1c level for each subject was measured at the beginning of this 12 week pre-intervention period (termed -12 time point) and compared with HbA1c pre-training level just before starting the intervention (0 time point). Participants' physicians were asked to maintain the subject on antihypertensive, lipid-altering, or glucose-lowering medications without alterations during the study unless medically necessary. When medication changes were deemed necessary, the subject was withdrawn from the study. Three subjects withdrew from the study during the 12 week pre-intervention period. The remaining twenty subjects were assigned to either the treadmill exercise (n = 10) or resistance exercise (n = 10) groups, matched by sex, age, and waist circumference, when possible (with an odd number of females, one group had 1 extra female). Subjects and the physical therapists could not be blinded to the study group, but study outcomes measurements and analyses were performed blinded.

### Exercise protocols

On the first visit, the exercise protocols were explained, and each subject completed a trial on the exercise machines. All exercise sessions were completed under the supervision of a physical therapist. The exercise regime consisted of sessions 3 times weekly, for 10 weeks. Each subject was given a training schedule that was maintained throughout the training period. In the isocaloric exercise protocols, subjects were asked to sit for 10 minutes pre exercise for acclimation. Heart rate, blood pressure, and blood glucose measurements were recorded pre and post exercise session. The training in both protocols progressed gradually in duration and intensity.

The treadmill training group exercised on the treadmill with continuous heart rate monitors (Polar Electro Oy, Kempele, Finland), which were used to adjust workload to achieve the target heart rate. Maximal heart rate was determined for all subjects in the study as the maximal heart rate during a single exercise bout to self-reported exhaustion. Participants progressed from 20 minutes per session at 60% of the maximum heart rate to 30 minutes per session at 75% of their maximum heart rate based on established protocols [31].

The resistance training group followed an individually monitored progressive resistance training program using multiple-station universal weight machines. Seven exercises were used for resistance training that encompassed knee and hip flexion/extension, shoulder flexion/extension, adduction/abduction, elbow flexion/extension and a chest press, based on previous publications [31]. Three sets of 8-10 repetitions were performed for all exercises. Subjects completed each exercise in a controlled rhythm, with a rest period of 2 minutes between sets. The intensity and duration of the resistance exercises were monitored by the physical therapist using heart rate monitors and adjusted [32] to follow the same heart rate progression as outlined above for the treadmill group. In this manner the average energy expenditure for both exercise groups was similar. The typical time required to complete the resistance exercises was 30-35 minutes, however it was dependent on the participant's heart rate. Before each session, subjects in both groups performed warm up exercises, consisting of stretching exercises for the major muscle groups. Participants were asked to report their perceived level of exertion during the peak performance of the exercises. There were no differences between the groups concerning their perceived exertion. No adverse events were reported by the participants during the study.

### Statistical analysis

Data were expressed as mean ± S.E. Repeated measure, one-way ANOVA was used to determine if there were differences among groups and then multiple comparison procedure (Tukey's tests) was used to isolate the differences. Statistical calculations were performed using Sigma Stat software (version 2.03). Differences were considered significant at p < 0.05. Figures illustrate means ± SE.

## Results

Attendance was verified through direct observation and maintenance of exercise logs demonstrating an 87% attendance rate. The subject's characteristics, collected prior to the exercise intervention, are shown in Table 2, illustrating no significant differences in the mean age, blood pressure, resting heart rate, waist circumference, or HbA1c values of the participants in the resistance and treadmill exercise groups.

### Heart rate and blood pressure were not altered by exercise training

The mean resting heart rate did not change over the course of the 10 week exercise program. Participants in the treadmill exercise program had a resting heart rate of 85 ± 1 beats per minute (bpm) prior to the initiation of the exercise program (week 0) and 85 ± 2 bpm at the completion of the 10 week program. In the resistive exercise group the initial resting heart rate was 86 ± 1 bpm and at the end of the training period the resting heart rate was 88 ± 1 bpm. Participant's heart rates were also monitored following their cool down after each exercise session. There were no differences between groups in the post-session heart rates.

The average resting blood pressure was not statistically different between the two intervention groups at the initiation of the study. The blood pressure was remarkably stable throughout the exercise program for both intervention groups. The treadmill group of subjects had a resting blood pressure of 135/84 mmHg at the initiation of the program (week 0), and 136/85 at the completion of the 10 weeks of aerobic exercise. The subjects receiving resistance training had an initial mean blood pressure of 138/85 mmHg and 10 weeks later their blood pressure was 136/84 mmHg.

### Exercise-induced reduction in plasma glucose levels

**Figure 1:**
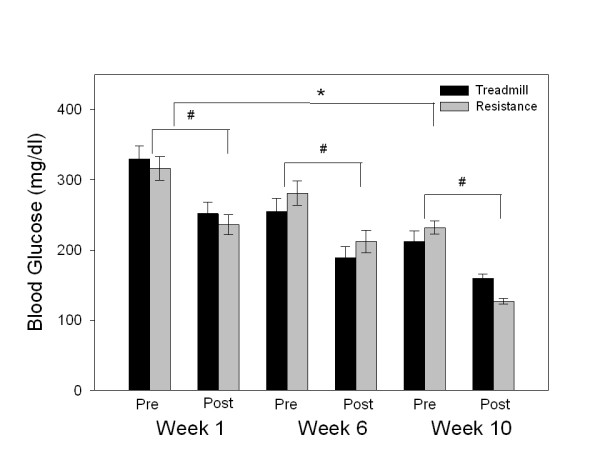
**Weekly mean pre- and post-exercise plasma glucose values for both groups shown for weeks 1, 6 and 10**. Both treadmill and resistance exercises caused a significant decrease in plasma glucose within each session (#, p < 0.05). Across the weeks, a decrease in pre-and post-exercise plasma glucose was measured with a significant decline from week 1 to 10 (*, p < 0.05).

Plasma glucose values were measured prior to, and at the end of each exercise session for both isocaloric intervention groups (Figure 1). Glucose values fell on average with each exercise session for both types of exercise (p < 0.05). Throughout the weeks of the training intervention, both pre- and post-exercise plasma glucose levels fell for participants in the treadmill and resistance exercise groups. This effect can best be illustrated in Figure 1 at weeks 1, 6, and 10. Repeated measures ANOVA indicated significant differences across the weeks (p < 0.0001). Pairwise multiple comparisons indicated that both treadmill and resistance exercise caused a significant reduction in the plasma glucose levels from week 1 to 10 (p < 0.05).

The mean week 10 post-exercise plasma glucose levels fell within the normal category of 140 mg/dl [33] or lower for the resistance exercise group. In fact, following resistance exercise at week 10, 80% of the participants had plasma glucose levels that fell within the normal recommendations after their exercise session. In contrast, only 20% of the participants reached normal glucose values (post-exercise session) in the treadmill group.

### Exercise-induced reduction in HbA1c

**Figure 2:**
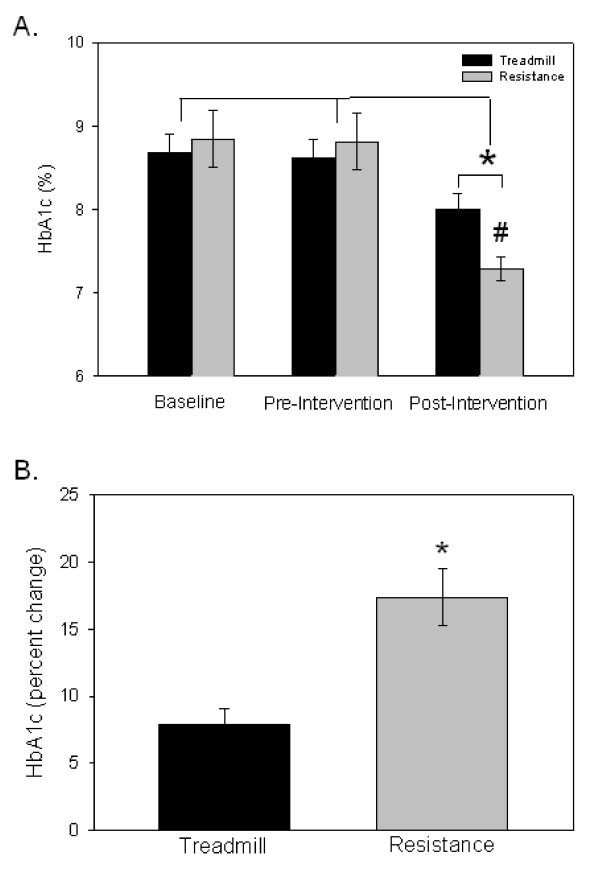
**HbA1c levels affected by exercise**. A) HbA1c values were collected 12 weeks prior to the initiation of the exercise program (Baseline), at the start of the exercise program (Pre-Intervention) and at the completion of the 10 weeks program (Post-Intervention). Ten week changes are denoted by * (p < 0.05). A difference between exercise groups is denoted by # (p < 0.008). B) Percentage change in the HbA1c levels from pre-intervention to post-intervention was greater in the resistance exercise group compared to the treadmill exercise group (*, p < 0.01).

There was no significant difference in the HbA1c levels during the pre-intervention period from baseline to 12 weeks later when the exercise protocol began (pre-intervention). The subjects all had HbA1c above the recommended level of 7%. The means at baseline were 8.7 and 8.9% for treadmill and resistance exercise groups, respectively. The range of HbA1c levels for all participants during the pre-intervention phase was from 7.4 to 10.4%. The HbA1c levels during this period did not correlate with the subject's age for either intervention group. Nor was there a statistical correlation between the baseline HbA1c level and waist circumference. During the pre-intervention period, the group assigned to resistive exercises showed a 0.03% reduction in HbA1c and the group assigned to treadmill exercise had a 0.05% reduction (Figure 2A). Neither of these differences were statistically significant.

In contrast, there was a significant reduction in HbA1c levels during the exercise interventions (p < 0.05; Figure 2A). Most interestingly, the group receiving resistance exercises had a significantly lower HbA1c level than the group receiving the treadmill intervention at the end of 10 weeks (p < 0.006). Not only were the absolute values significantly different, but the percent change in the HbA1c levels were different between the two groups (Figure 2B; p < 0.01). While aerobic exercise did lower the HbA1c levels significantly (p < 0.05), it did not bring them into the target range (< 7%). None of the participants in the treadmill group reached a HbA1c level of 7% or less, while 40% of the participants in the resistance exercise group reached target HbA1c levels. There was no statistically significant correlation between the reduction in HbA1c levels and the age of the participant or their initial waist circumference.

## Discussion

Exercise is generally recommended for people with type 2 diabetes. Several studies have evaluated the effects of exercise training on glycemic control. However, the beneficial effects of different types of exercise on glycemic control have not been well differentiated. The present study was designed to compare the beneficial effects of resistance versus aerobic exercise in age- and sex-matched groups of adults with type 2 diabetes. The results of this study showed clearly that both treadmill and resistance exercise have a positive effect on glucose control in people with type 2 diabetes, as noted from the steady reduction in the blood glucose readings taken during each intervention session (Figure 1), and the change in HbA1c levels at the completion of the exercise program (Figure 2). Most important was the fact that the subjects receiving a progressive resistance exercise program showed a significantly greater improvement in HbA1c levels over the 10 week period compared to those undergoing treadmill exercise (Figure 2B). The resistance training group showed a reduction in the HbA1c values of 18% as compared to an 8% reduction in the treadmill exercise group. One must keep in mind the conclusion by the UKPDS-33 that even an 11% reduction in HbA1c results in a 25% reduction in complications associated with the disease [2]. Accordingly, a doubling of the decline in the HbA1c level with a progressive resistance exercise routine when compared to the treadmill protocol, as we demonstrated in this study, is clinically important.

In this study we maintained similar energy expenditure in both groups by monitoring heart rate and maintaining a range of 60-75% maximal heart rate for both groups. While heart rate is only an estimation of energy exerted, it has been validated as an accurate reflection of energy expenditure [34]. Heart rate can be monitored for every exercise training session and thus control the level of training. Other methods such as direct or indirect calorimetry are expensive and too cumbersome to use for each training session. Dropout of subjects would have likely been much higher, had we required calorimetry measurements for each session. It is important to note that using heart rate as a way to control energy expenditure is complicated by the fact that other factors such as emotions can alter heart rate. For this reason, we also asked for the subject's perception concerning their exertion.

A few studies have looked at the combined effect of aerobic and resistance exercises [35-37]. In studies where resistance and aerobic exercise is combined, positive effects are noted, but the absolute reduction in HbA1c levels have not been shown to be any better than those we report with resistance exercise alone. Two studies reported reductions in the HbA1c values of 0.6 with combined aerobic and resistance exercises of 8 weeks or more [35,38]. Tokmakidis showed a reduction of 0.8 percentage points and Sigal et al had a reduction of 0.9 percentage points in HbA1c with combined aerobic and resistance exercises [30,36]. We report a decrease of 1.6 percentage points over the course of a 10 week resistance training program. More in line with our study, Christos et al showed a reduction of 1.2 percentage points with 4 months of combined exercise. Our large drop in percentage points is likely due to the fact that the subjects enrolled in this study had baseline HbA1c levels that were dramatically higher than those reported in the other studies. For subjects in our study, the mean baseline HbA1c level was 8.8 ± 0.9% while Sigal et al. reported 7.5 ± 1.5% [30]. For the subjects involved in this study, the resistance protocol utilized appears to provide all of the advantages of combined aerobic/resistance exercise programs shown by other researchers.

Our findings are in agreement with Cauza et al, showing a greater improvement in blood glucose control following 4 months of strength training as opposed to endurance exercise [39]. In fact, they showed no significant reduction in mean blood glucose levels, as monitored by continuous glucose monitors in the aerobic endurance training group. In this study we examined both the immediate pre- and post-exercise blood glucose levels and the long-term implications using the HbA1c levels. The non-fasting blood glucose levels and the HbA1c levels all declined more with strength training than aerobic exercise. The non-fasting glucose levels are important as other authors have shown that they are more sensitive to the beneficial effects of exercise, whereas fasting glucose values are not associated with physical activity [40]. It is noteworthy that the dramatic changes in blood glucose were not associated with any changes in the resting heart rate or blood pressure in either group. A recent meta-analysis also failed to find a significant exercise-induced change in heart rate or blood pressure [13]. Another factor is that the baseline blood pressure values for the subjects in the current study were already tightly controlled prior to the exercise intervention.

Given that the etiology of type 2 diabetes consists of insulin resistance within skeletal muscle, perhaps it is not surprising that resistance exercises had more of a positive effect on glucose control than aerobic exercise. Resistance exercises have been shown to have a significant impact on insulin sensitivity in people with type 2 diabetes [41]. In addition, vascular endothelial cell function improved with 14 months of resistance training in people with type 2 diabetes [42]. Caution should be noted as not all resistance training appears to be beneficial. A home-based training program using resistance exercise bands was not sufficient to improve glycemic control [43].

In summary, both resistance and aerobic exercise protocols were effective in reducing pre- and post-exercise blood glucose levels and HbA1c levels, but resistance exercise produced a more significant reduction in HbA1c level as compared to treadmill exercise. We propose that an optimal exercise program for individuals with diabetes should include a resistance training component to be effective in improving the overall metabolic profile, and thus reduce the risk for long term diabetic complications in type 2 diabetes.

## Competing interests

The authors declare that they have no competing interests.

## Authors' contributions

SW assisted in the design and collected the outcomes measures. MA designed the study, analyzed the data and drafted the manuscript. AA and MM recruited subjects and implemented the exercise protocols for both groups. IVS assisted in statistical analysis of the data and drafting of the manuscript. LN created figures and drafted the manuscript involving the interpretation of the data. LSB assisted in design, completed statistical analysis and oversaw the completion of the manuscript. All authors read and approved the final manuscript.
